# Multiplex functional connectome graph transformer for cognitive vulnerability in dialysis-treated chronic kidney disease

**DOI:** 10.3389/fpsyt.2026.1906443

**Published:** 2026-08-12

**Authors:** Junjie Zhai, Jiachang Pang, Xiangdong Lei, Kexu Yan, Jingren Zhou

**Affiliations:** School of Information Science and Engineering, Chongqing Jiaotong University, Chongqing, China

**Keywords:** chronic kidney disease, cognitive impairment, graph transformer, multiplex connectome, neuroimaging, resting-state functional magnetic resonance imaging

## Abstract

**Objective:**

Chronic kidney disease (CKD) requiring maintenance dialysis is accompanied by cognitive vulnerability and distributed brain-network dysfunction. This study evaluated whether complementary resting-state functional magnetic resonance imaging (rs-fMRI) connectome views could support subject-level characterization of CKD-related neurocognitive brain dysfunction.

**Methods:**

We analyzed a de-identified rs-fMRI dataset of 100 participants, including 52 patients with CKD receiving maintenance dialysis and 48 normal controls (NCs). The CKD group had lower Mini-Mental State Examination and Montreal Cognitive Assessment scores. AAL116 regional time series were represented as three 116 × 116 graph views: Pearson correlation, sparse representation, and Granger causality mapping. We developed MTGAF, a multiplex graph transformer combining modality-specific graph encoding, graph-transformer refinement, disease-aware readout, cross-modal attention, and adaptive fusion. Performance was evaluated with participant-level stratified 5-fold cross-validation and compared with BrainNetTF, BrainGNN, SVM, Random Forest, single-modality variants, and additional component-ablation variants. Robustness was assessed using pooled confusion matrices, bootstrap confidence intervals, calibration summaries, paired comparisons, and ROI importance analysis.

**Results:**

MTGAF achieved accuracy 0.8800 ± 0.0678, macro-F1 0.8796 ± 0.0680, and AUC 0.9297 ± 0.0437. The pooled confusion matrix contained 42 true negatives, 46 true positives, 6 false positives, and 6 false negatives. Bootstrap 95% CIs were 0.8100–0.9400 for accuracy, 0.8095–0.9396 for macro-F1, and 0.8696–0.9770 for pooled AUC. The full PC–SR–GCM model achieved higher accuracy and macro-F1 than single-modality variants. ROI importance highlighted cingulate, temporoparietal, sensorimotor, visual-association, frontostriatal, posterior cingulate, and temporal-association systems.

**Conclusions:**

Multiplex modeling of complementary connectome information provides signals for characterizing CKD-related neurocognitive brain-network dysfunction. MTGAF is a research-stage imaging-AI framework for individualized brain-risk characterization in dialysis-treated CKD and should be further evaluated in larger multi-center cohorts.

## 1 Introduction

Chronic kidney disease (CKD) is a progressive systemic disorder defined by persistent abnormalities in kidney structure or function and may ultimately require dialysis or transplantation (1). Its clinical burden extends beyond renal failure and includes cognitive, behavioral, and functional consequences that are relevant to neuropsychiatric care. Studies have shown that CKD is associated with cognitive impairment, reduced treatment adherence, impaired self-management, frailty, poorer quality of life, and adverse long-term outcomes (2–8). The risk of cognitive decline appears to increase with renal dysfunction, vascular comorbidity, and dialysis-related exposure (9–12). Structural and multimodal neuroimaging studies, including the Brain in Kidney Disease study and later kidney-brain crosstalk work, further indicate that kidney dysfunction is accompanied by measurable brain abnormalities (13, 14). These findings establish CKD-related neurocognitive brain dysfunction as a clinically meaningful problem, while validated imaging-derived markers for individualized characterization 44 remain limited.

Resting-state functional magnetic resonance imaging (rs-fMRI) offers a noninvasive way to examine CKD-related brain-network alterations. Classical CKD and end-stage renal disease studies have reported abnormal default mode network connectivity, altered large-scale functional organization, disrupted structural-functional coupling, and graph-theoretical abnormalities in hemodialysis, peritoneal dialysis, and cognitively impaired CKD cohorts (15–24). More recent studies have expanded this literature to dynamic functional connectivity, interhemispheric coordination, neurovascular coupling, cognitive prediction, and blood-oxygen-level-dependent cerebrospinal-fluid coupling (25–34). However, much of this work remains centered on group-level comparisons or correlation analyses. Such findings are valuable for mechanism discovery, while the subject-level prediction question remains less explored.

A related methodological gap is that most rs-fMRI prediction approaches rely on a single functional connectivity representation, most commonly Pearson correlation. This simplification is convenient, although complementary disease-relevant information may remain underused. CKD involves vascular dysfunction, metabolic stress, inflammation, and dialysis-related physiological burden, and these processes may influence brain networks through partially distinct patterns of synchrony, sparse dependency, and temporal association. Pearson correlation (PC) captures undirected synchronous coupling, sparse representation (SR) emphasizes informative sparse dependencies, and Granger causality mapping (GCM) describes directed temporal associations between regions (35). Multimodal neuroimaging, radiology-AI, and data-fusion studies have repeatedly shown that disease information may be distributed across complementary representations and can be weakened when compressed into one view (36–39). Related psychiatric neuroimaging work has also used multiple functional connection patterns to improve disorder identification, supporting the broader methodological premise that complementary connectome views can carry nonredundant predictive information (40). Recent Meta-Radiology articles likewise illustrate this broader imaging-AI direction through complex brain-network representation, multimodal MRI graph-attention prediction in psychiatric imaging, constrained multiview representation learning, and foundation-model-assisted fusion for neurodegenerative diagnosis (41–44). These considerations motivate a multiplex brain-network representation rather than a single-matrix model.

Graph-based deep learning is well suited to this problem because it preserves the topology of brain networks while enabling subject-level prediction (45). In neuroimaging, graph convolutional and related models have been applied to classification, prediction, and interpretation across Alzheimer’s disease, functional-connectivity modeling, hierarchical brain-disorder analysis, amyloid prediction, graph-signal transformation, infant connectomics, and interpretable fMRI learning (46–52). Attention-based architectures further provide a way to model long-range dependencies in graph-structured data (53, 54). Nevertheless, for CKD rs-fMRI, it remains unclear whether a graph-transformer framework that explicitly fuses multiple connectivity views can improve individualized identification and yield interpretable disease-related brain-network patterns.

To address these gaps, we developed MTGAF, a multiplex functional-connectome graph transformer with adaptive cross-modal fusion, designed to characterize CKD-related alterations in brain-network organization at the individual-subject level. MTGAF represents each participant using three complementary connectome views: Pearson correlation (PC), sparse representation (SR), and Granger causality mapping (GCM). These views capture synchronized activity, sparse dependency structure, and directed temporal associations, respectively. The model further combines modality-specific graph encoding, graph-transformer refinement, disease-aware readout, pairwise cross-modal attention, and adaptive fusion, allowing it to learn both within-view topology and between-view complementarity. In this study, the direct prediction task was the discrimination of CKD patients from normal controls, rather than the diagnosis of individual cognitive impairment. Cognitive vulnerability was used as clinical context, supported by group-level differences in MMSE and MoCA scores in the source cohort. We therefore evaluated MTGAF by comparing its subject-level CKD classification performance with neural-network and conventional machine-learning baselines, assessing the contribution of the joint PC–SR–GCM representation through ablation analyses, and examining whether model-derived regional importance patterns were consistent with CKD-related cognitive and brain-system alterations.

## 2 Materials and methods

### 2.1 Study design and dataset

The dataset used in this study was derived from the cohort reported by Zhang et al. in their rs-fMRI study (55). The final modeling dataset included 100 participants with binary labels, comprising 52 individuals with CKD receiving maintenance dialysis and 48 normal controls (NCs). The binary class label used for model training was CKD versus NC status. Cognitive scores were used for clinical characterization rather than as prediction labels, so model outputs are interpreted as CKD-versus-NC classification rather than individual cognitive diagnosis. Among the 52 CKD participants, 27 underwent peritoneal dialysis and 25 underwent hemodialysis.

All participants were recruited from Liangxiang Teaching Hospital of Capital Medical University. Patients met the clinical criteria for end-stage renal disease (ESRD) and received maintenance dialysis. Normal controls were recruited during the same period. In the original cohort, demographic information, dialysis status, vascular/metabolic comorbidities, laboratory measurements, and cognitive scores were collected as part of the clinical assessment (55). The present analysis used de-identified secondary imaging matrices derived from that cohort.

The rs-fMRI data were acquired on a Siemens Magnetom Skyra 3.0T scanner equipped with a 32-channel phased-array head coil using a single-shot gradient-echo echo-planar imaging sequence. The acquisition parameters were as follows: repetition time (TR) = 2,000 ms, echo time (TE) = 30 ms, flip angle = 90°, matrix size = 64 × 64, field of view (FOV) = 224 × 224 mm, slice thickness = 4 mm, 36 slices, and 180 volumes, with fat suppression enabled and a parallel imaging factor of 2. During scanning, participants were instructed to keep their eyes closed, remain still, stay relaxed, and stay awake (55).

For each participant, the available imaging data included: (1) a 170 × 116 rs-fMRI time-series matrix, where 170 denotes temporal samples and 116 denotes brain regions of interest from the Automated Anatomical Labeling 116-region (AAL116) parcellation used in the source dataset; and (2) three precomputed 116 × 116 connectivity matrices corresponding to the PC, SR, and GCM views. During data processing, each participant’s time-series matrix was transposed to 116 × 170, such that each AAL116 brain region was represented by its regional blood-oxygen-level-dependent time series. These regional signals were normalized using z-score normalization within each subject to reduce inter-regional variability before model training. All normalization steps were performed independently within each subject, thereby avoiding information leakage across data splits.

### 2.2 Multimodal functional network representation

Each participant was represented by three parallel functional brain networks derived from the same rs-fMRI acquisition. The first network was based on Pearson correlation (PC) and was intended to describe undirected temporal synchrony between brain regions. The second network was based on sparse representation (SR) and was used to emphasize the most informative conditional dependency structure while reducing redundant connections. The third network was derived from Granger causality modeling (GCM) and was intended to characterize directed temporal association between regions (35). In this way, each participant contributed three complementary descriptions of the same functional brain organization rather than a single connectivity matrix.

The rs-fMRI time-series matrix for each participant contained 116 brain regions and 170 temporal observations per region after transposition for analysis. Thus, each node in the graph corresponded to one brain region, and the node feature vector corresponded to that region’s normalized rs-fMRI time course. We chose to preserve the three connectivity modalities separately because CKD-related brain dysfunction may be expressed differently across correlation strength, sparse dependency structure, and directional information flow. Keeping these modalities separate before fusion allowed the model to learn modality-specific information first and integrate it only at a later stage.

Formally, let $X=[x_{1},x_{2},\ldots ,x_{N}]\in {\mathbb{R}}^{T\times N}$ denote the subject-specific rs-fMRI matrix, where *N* = 116 is the number of brain regions and *T* = 170 is the number of temporal observations. For each participant, 3 subject-specific adjacency matrices, *A*_PC_, *A*_SR_, and *A*_GCM_, were constructed for subsequent graph analysis.

For Pearson’s correlation (PC), the connectivity weight *w_ij_* between the *i*-th and *j*-th ROIs was computed as the Pearson correlation coefficient of their regional time series *x_i_* and *x_j_* (56). The three functional connectivity representations are mathematically defined in Equations 1–3:

(1) $$w_{ij}=\frac{{\left(x_{i}-{\bar{x}}_{i}\right)}^{T}(x_{j}-{\bar{x}}_{j})}{\sqrt{{\left(x_{i}-{\bar{x}}_{i}\right)}^{T}(x_{i}-{\bar{x}}_{i}){\left(x_{j}-{\bar{x}}_{j}\right)}^{T}(x_{j}-{\bar{x}}_{j})}},$$

where ${\bar{x}}_{i}$ denotes the mean of the BOLD signal in the i-th ROI. The resulting matrix $A_{\text{PC}}=[w_{ij}]\in {\mathbb{R}}^{N\times N}$ described undirected temporal synchrony between regions.

For sparse representation (SR), an *ℓ*_1_-regularized sparse regression model was used to estimate conditional dependence between ROIs (57):

(2) $$\underset{W_{i}}{\min}\;x_{i}-∥\sum_{j\neq i}W_{ij}x_{j}{∥}_{2}^{2}+\lambda \sum_{j\neq i}∥W_{ij}{∥}_{1},$$

where *W_ij_* represents the sparse connectivity weight from ROI *j* to ROI *i*, and *λ* controls the regularization strength. The regularization parameter was set to *λ* = 0.1 in the primary analysis. The estimated coefficients were assembled into the SR adjacency matrix $A_{\text{SR}}=[W_{ij}]\in {\mathbb{R}}^{N\times N}$.

For Granger causality mapping (GCM), directed temporal associations between ROIs were quantified using Granger causality analysis with a first-order autoregressive model (35, 56). The first-order model was used to limit parameter burden in the short rs-fMRI time series and to keep the GCM view computationally consistent across participants. Given the temporal resolution and length of rs-fMRI data, the GCM matrix was interpreted as a directed temporal-association view rather than as a causal mechanistic endpoint:

(3) $$F_{x_{j}\to x_{i}}=\ln\;\frac{\sum_{t=1}^{T}\epsilon_{i,t}^{2}}{\sum_{t=1}^{T}\eta_{i,t}^{2}},$$

where *ϵ_i,t_* and *η_i,t_* denote the residuals at time *t* from the restricted and unrestricted regression models for ROI *i*, respectively. Larger values of *F_xj_*→*_xi_* indicate stronger directed temporal association from region *j* to region *i*. The resulting directed adjacency matrix was denoted by $A_{\text{GCM}}\in {\mathbb{R}}^{N\times N}$.

### 2.3 Graph construction and edge selection

Let $A^{\left(m\right)}\in {\mathbb{R}}^{N\times N}$ denote the adjacency matrix for modality m∈{PC,SR,GCM}, where N=116 is the number of brain regions. For each node i, a modality-specific top- k operator $alT_{k_{m}}(\cdot )$ was applied row-wise to retain the strongest connections and generate a sparse adjacency matrix ${\tilde{A}}^{\left(m\right)}$. In the final MTGAF configuration, *k*_PC_ = 20, *k*_SR_ = 15, and *k*_GCM_ = 10. Edge selection was performed according to the absolute magnitude of each connection, while the original signed weights were retained after selection for PC and SR. GCM values were retained as nonnegative directed temporal-association weights. Self-connections were removed before top-*k* selection, and self-loops were then added during graph convolution to preserve each node’s own feature contribution. PC and SR were treated as undirected functional networks, whereas GCM was retained as a directed graph reflecting asymmetric temporal association between regions. A modality-specific sparsification strategy was adopted to balance information retention and noise reduction across the three connectivity definitions.

After edge selection, edge weights were scaled by the maximum absolute edge value within each graph when nonzero values were present. This step was used to place different participants and modalities on a comparable numerical scale and to improve training stability. Therefore, each participant entered the model as a triplet of synchronized graphs sharing the same node features but differing in their connectivity patterns.

### 2.4 Model development

MTGAF was developed as a three-branch deep learning framework in which the PC, SR, and GCM networks of the same participant were analyzed simultaneously. Each branch followed the same graph-encoding design so that the three connectivity views could first be modeled within their own topological spaces before cross-modal integration. This design was selected to preserve modality-specific information and to avoid reducing the participant’s functional organization to a single connectivity matrix at the input stage.

In the first stage, each modality-specific graph was processed by a graph convolutional encoder, which transformed the 170-dimensional regional feature vectors into a 48-dimensional latent representation. The graph convolution stage was intended to capture local topological structure and neighborhood-level signal interactions within each functional network. Positive and negative edges could be treated separately, and the final signed top-*k* setting retained the selected adjacency values for graph construction.

In the second stage, the graph-convolution representation from each modality was refined by a graph transformer module with 2 attention heads and 1 transformer layer (54). In practical terms, this module allowed the model to weigh connections differentially and to model longer-range inter-regional dependencies that may not be fully captured by local graph convolution alone. Thus, each modality produced a node-level latent representation that incorporated both local graph structure and more global dependency information.

In the third stage, node-level features were converted into participant-level modality summaries using a disease-aware readout and learnable mixed pooling. The disease-aware readout learned node weights that emphasized brain regions contributing more strongly to the CKD classification objective. Mixed pooling combined global mean pooling and global max pooling, allowing the model to retain both distributed network information and highly responsive regional features.

To model interactions among modalities, the pooled PC, SR, and GCM representations were compared in a pairwise manner through a bidirectional attention mechanism. In functional terms, each modality was allowed to attend to features from the others, yielding three interaction features corresponding to PC-SR, PC-GCM, and SR-GCM. These interaction features were intended to quantify whether patterns observed in one connectivity space reinforced or modified the information carried by another connectivity space. The original modality-specific pooled features and the interaction features were then passed to a dual-stream adaptive fusion block, which learned how much weight should be assigned to direct modality information and how much should be assigned to cross-modal interaction information.

**Figure 1:**
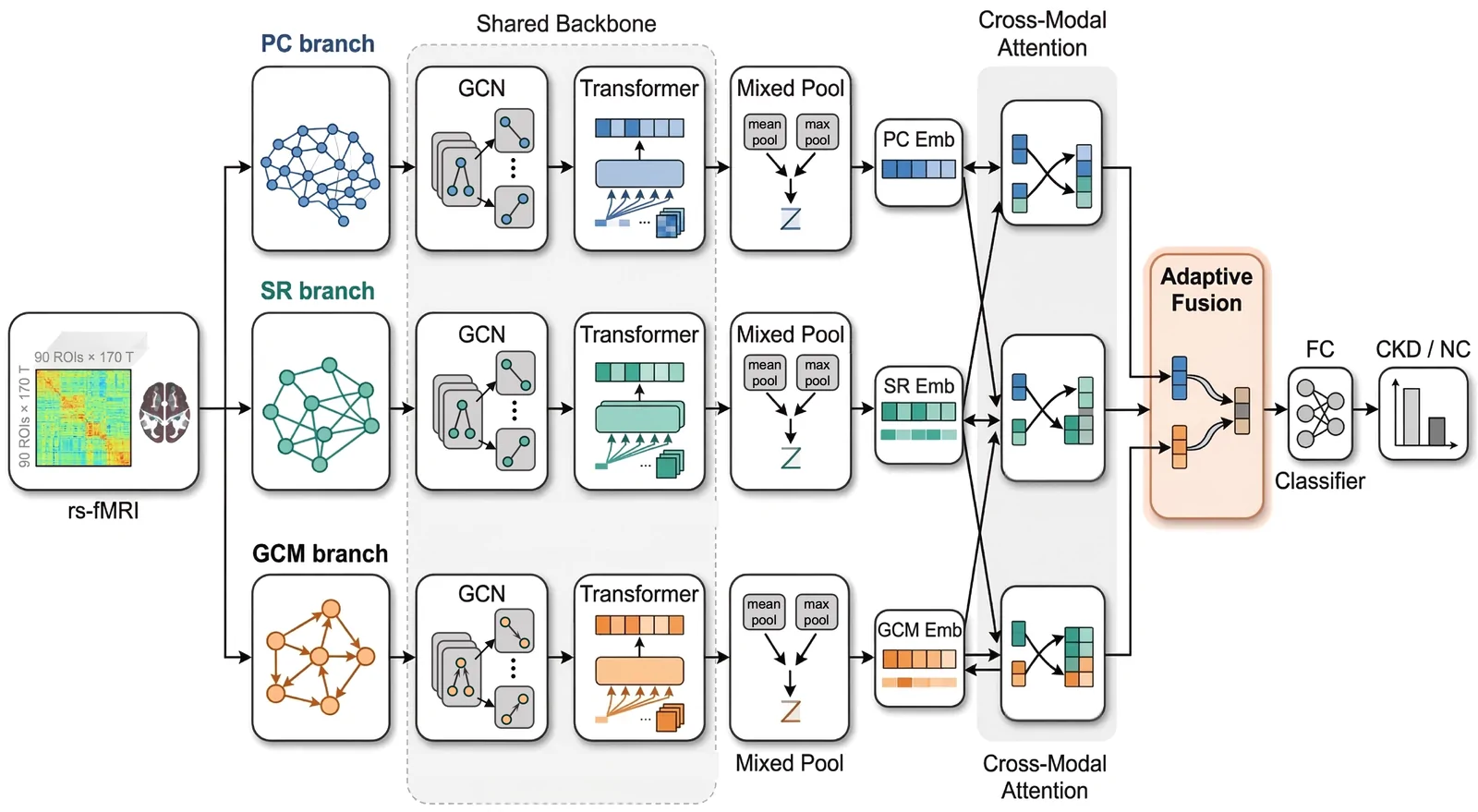
Overview of the MTGAF framework. Each participant contributed one resting-state functional magnetic resonance imaging feature matrix and three synchronized functional connectome views: PC for temporal synchrony, SR for sparse dependency structure, and GCM for directed temporal association. The three graph branches passed through modality-specific graph-convolution and graph-transformer modules, mixed pooling, pairwise cross-modal attention, adaptive fusion, and a fully connected classifier for CKD versus NC identification.

The fused representation was finally passed through a two-layer fully connected classifier with batch normalization, rectified linear activation, and dropout to generate the probability of CKD versus NC. The overall model architecture is illustrated in **Figure 1**.

### 2.5 Use of multimodal information during model training

The multimodal strategy was implemented at the participant level. During each forward pass, the model received the three synchronized graphs belonging to the same participant together with a single binary class label. Accordingly, the model was trained end-to-end as one integrated classifier rather than as three independent classifiers followed by majority voting or score averaging. This design allowed multimodal information to influence the feature-learning process itself, not only the final decision stage.

From a methodological perspective, the graph convolution and transformer components learned modality-specific representations, whereas the bidirectional attention and adaptive fusion components learned how the three modalities should be combined before classification. This architecture was intended to preserve complementary information from each connectivity definition while reducing the risk that one modality would dominate the prediction by itself.

### 2.6 Training procedure

Deep-learning models were trained using the cross-entropy objective and the Adam optimizer (learning rate 5 × 10^−4^; weight decay 1 × 10^−5^; batch size 32). The final MTGAF configuration used a hidden dimension of 48, 2 transformer attention heads, 1 transformer layer, transformer dropout of 0.4, classifier dropout of 0.5, and signed top-*k* graph construction with *k*_PC_ = 20, *k*_SR_ = 15, and *k*_GCM_ = 10. Hyperparameters were selected during method development based on training-fold behavior and model stability and then fixed for the reported 5-fold evaluation; test-fold samples were not used to update model settings. The primary model contained 201,878 trainable parameters. Linear layers were initialized with Kaiming or Xavier initialization according to their role in the encoder or classifier, transformer projection weights were initialized from a zero-mean normal distribution, and batch-normalization scale and bias parameters were initialized to 1 and 0, respectively. The base random seed was 396. Training was run for up to 300 epochs with a ReduceLROnPlateau scheduler, dropout regularization, and early stopping.

For methodological comparison, we evaluated MTGAF, BrainNetTF, BrainGNN, support vector machine (SVM), and Random Forest classifiers using the same participant-level 5-fold evaluation framework. BrainNetTF and BrainGNN were included as neural connectome baselines, whereas SVM and Random Forest served as conventional machine-learning baselines. To make the multiplex comparison fair, all PC, SR, and GCM matrices from the same participant were kept together during data splitting and were not treated as independent samples. The same fold assignments were used for all comparator models, and model-specific settings were kept fixed across folds after method development. BrainNetTF was adapted by constructing a multimodal connection-profile input for each participant, in which each ROI token contained the concatenated PC, SR, and GCM connection profiles before transformer encoding and cluster readout. BrainGNN was adapted with 3 modality-specific graph-convolution branches followed by ROI-selection pooling and a modality-gating classifier. For SVM and Random Forest, the PC, SR, and GCM connectivity matrices were vectorized and concatenated into participant-level feature vectors before classifier training. Thus, baseline comparisons used the same participants, labels, and cross-validation partitions, while differing in how each model encoded the multiplex connectome information.

Beyond the main model comparison and single-modality ablations, we performed component ablation experiments to examine how model capacity and major architectural modules affected classification performance. The reduced-capacity MTGAF-small variant retained the same PC, SR, and GCM inputs and overall fusion framework, but used a hidden dimension of 24, 1 transformer head, and 1 transformer layer. Additional variants removed the graph-transformer block, removed pairwise cross-modal attention, replaced adaptive fusion, or removed disease-aware readout. These analyses evaluated the contribution of each major structural component to model behavior.

### 2.7 Data split and evaluation

Model performance was evaluated using stratified 5-fold cross-validation at the participant level. Each participant contributed synchronized PC, SR, and GCM graphs and appeared in one test fold for each model. Performance was summarized using accuracy, precision, recall, macro-F1, and AUC. Performance estimates are reported as mean ± standard deviation across 5 folds.

For robustness assessment, cross-validation models were used to calculate confusion matrices, bootstrap confidence intervals, calibration summaries, and paired comparisons.

Robustness analyses were performed from the participant-level out-of-fold predictions. For each model, pooled test-set confusion matrices were calculated across the 5 folds, and participant-level nonparametric bootstrap resampling with 2,000 resamples was used to estimate percentile 95% confidence intervals for accuracy, macro-precision, macro-recall, macro-F1, AUC, Brier score, and expected calibration error. Calibration was assessed using the Brier score and expected calibration error computed from the same out-of-fold predictions. Paired comparisons between MTGAF and each comparator were calculated using participant-matched predictions, with bootstrap confidence intervals for accuracy, macro-F1, and AUC differences and exact McNemar tests for binary error differences.

Baseline demographic and clinical variables were summarized descriptively. Continuous variables are presented as mean ± standard deviation, and categorical variables are presented as counts. The source cohort reported group-comparison *p*-values for baseline variables; continuous variables were evaluated using two-sample group comparisons and sex distribution using a categorical group comparison. In the present manuscript, these *p*-values characterize baseline group balance and cognitive-score differences in the source cohort; they were not used for feature selection or model training.

The model optimization objective and performance metrics are defined in Equations 4–8.

(4) $$L=-\frac{1}{M}\sum_{n=1}^{M}\sum_{c=1}^{C}y_{n,c}\log\;{\hat{p}}_{n,c},$$

where *M* is the batch size, *C* = 2 is the number of classes, $y_{n,c}$ is the one-hot reference label, and ${\hat{p}}_{n,c}$ is the predicted probability for class *c*. Let *TP*, *TN*, *FP*, and *FN* denote the numbers of true positives, true negatives, false positives, and false negatives, respectively. Accuracy, precision, recall, and F1 score were calculated as

(5) $$\text{Accuracy}=\frac{TP+TN}{TP+TN+FP+FN},$$

(6) $$\text{Precision}=\frac{TP}{TP+FP},$$

(7) $$\text{Recall}=\frac{TP}{TP+FN},$$

(8) $$\text{F}1=\frac{2\cdot \text{Precision}\cdot \text{Recall}}{\text{Precision}+\text{Recall}}.$$

Because the reported task was binary classification (CKD versus NC), macro-precision, macro-recall, and macro-F1 were obtained by averaging the corresponding class-wise values across the 2 classes.

## 3 Results

### 3.1 Baseline characteristics

A total of 100 participants were included in the final modeling dataset, comprising 48 normal controls (NC) and 52 patients with chronic kidney disease (CKD) receiving maintenance dialysis. The baseline demographic and clinical characteristics are summarized in **Table 1**. There was no significant difference in age (55.3 ± 9.5 vs. 57.8 ± 11.2 years, *p* = 0.236) or sex distribution (*p* = 0.584) between the NC and CKD groups. However, compared with the NC group, CKD patients exhibited lower cognitive scores in the source cohort, including the Mini-Mental State Examination (MMSE) (28.5 ± 1.2 vs. 23.8 ± 3.2, *p <* 0.001) and the Montreal Cognitive Assessment (MoCA) (27.8 ± 1.6 vs. 24.3 ± 2.5, *p <* 0.001). The mean disease duration for the CKD group was 64.2 ± 58.7 months.

**Table 1:** Baseline demographic and clinical characteristics of the participants.

Characteristic	NC (*n* = 48)	CKD (*n* = 52)	*p*-value
Age (years)	55.3 ± 9.5	57.8 ± 11.2	0.236
Sex (Male/Female)	30/18	28/24	0.584
MMSE score	28.5 ± 1.2	23.8 ± 3.2	*<* 0.001
MoCA score	27.8 ± 1.6	24.3 ± 2.5	*<* 0.001
Disease duration (months)	—	64.2 ± 58.7	—

### 3.2 Classification performance

**Figure 2:**
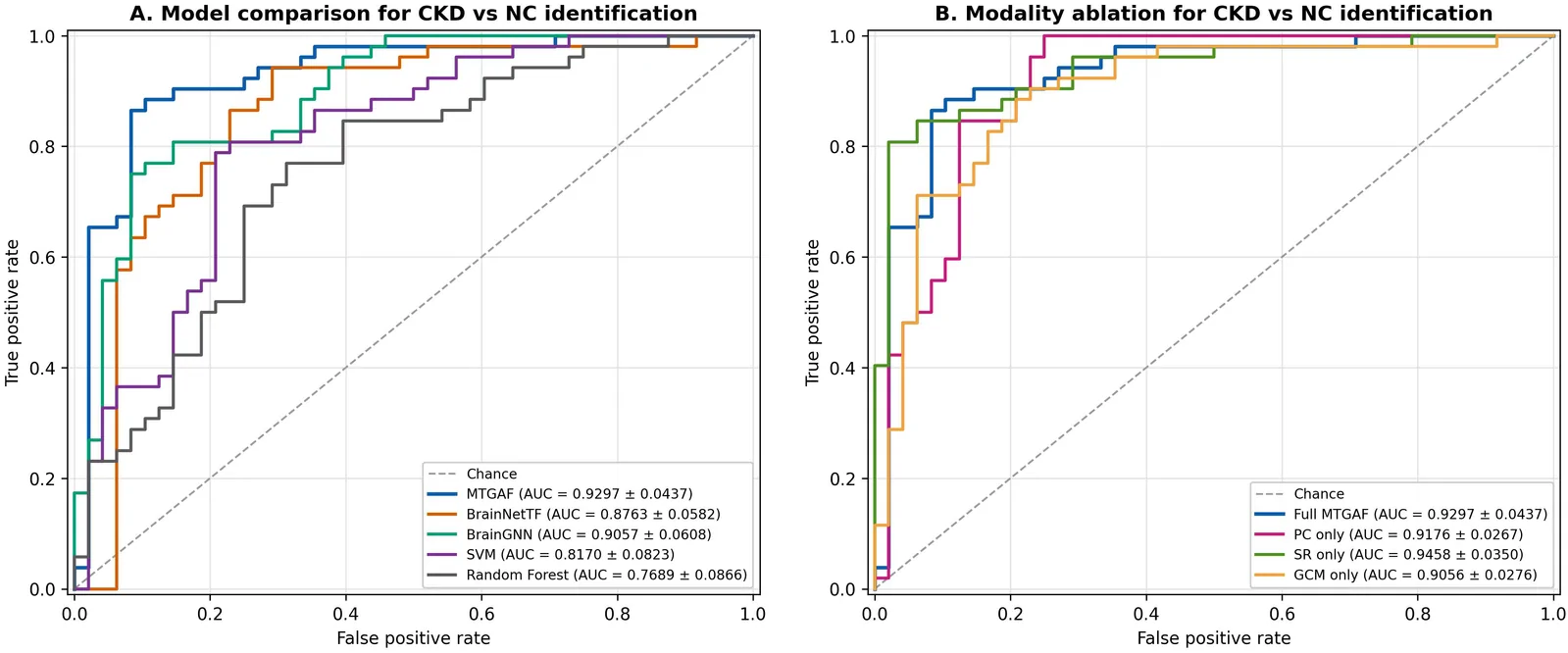
ROC summaries for CKD versus normal control identification. **(A)** compares MTGAF with BrainNetTF, BrainGNN, SVM, and Random Forest. **(B)** compares the full PC–SR–GCM MTGAF with single-modality variants. The displayed curves were generated from pooled out-of-fold test predictions to show the overall ROC shape, whereas the legend AUC values report the mean ± standard deviation of fold-wise AUC values across the 5 cross-validation folds.

Across the 5 cross-validation folds, MTGAF achieved a test accuracy of 0.8800 ± 0.0678, macro-F1 of 0.8796 ± 0.0680, and AUC of 0.9297 ± 0.0437 for CKD versus NC classification. Fold-wise test accuracy ranged from 0.75 to 0.95, indicating generally stable performance across test partitions with measurable fold-to-fold variation. To support interpretation in the present single-center cohort, performance was further assessed using out-of-fold predictions, bootstrap confidence intervals, calibration summaries, reduced-capacity analysis, and component ablations. The corresponding ROC summaries for model comparison and modality ablation are shown in **Figure 2**.

### 3.3 Comparison with baseline models and ablation variants

To evaluate whether the observed CKD classification performance depended on the specific MTGAF design, we compared MTGAF with BrainNetTF, BrainGNN, SVM, and Random Forest classifiers. **Table 2** reports the 5-fold cross-validation results for each model.

**Table 2:** Five-fold cross-validation performance comparison for CKD classification.

Model	Accuracy	Precision	Recall	F1-score	AUC
**MTGAF**	**0**.**8800* ± *0**.**0678**	**0**.**8833* ± *0**.**0691**	**0**.**8814* ± *0**.**0678**	**0**.**8796* ± *0**.**0680**	**0**.**9297* ± *0**.**0437**
BrainNetTF	0.8000 ± 0.1000	0.8173 ± 0.1030	0.7970 ± 0.1020	0.7949 ± 0.1026	0.8763 ± 0.0582
BrainGNN	0.7600 ± 0.0583	0.7624 ± 0.0594	0.7581 ± 0.0584	0.7579 ± 0.0583	0.9057 ± 0.0608
SVM	0.7700 ± 0.0812	0.7762 ± 0.0912	0.7677 ± 0.0843	0.7670 ± 0.0834	0.8170 ± 0.0823
Random Forest	0.7200 ± 0.1122	0.7208 ± 0.1120	0.7159 ± 0.1124	0.7161 ± 0.1128	0.7689 ± 0.0866

Bold formatting identifies the best-performing result for each metric.

Relative to these baselines, MTGAF achieved the numerically highest test accuracy and macro-F1, with higher mean values than BrainNetTF (0.8000 ± 0.1000 accuracy and 0.7949 ± 0.1026 macro-F1), BrainGNN (0.7600 ± 0.0583 accuracy and 0.7579 ± 0.0583 macro-F1), SVM (0.7700 ± 0.0812 accuracy and 0.7670 ± 0.0834 macro-F1), and Random Forest (0.7200 ± 0.1122 accuracy and 0.7161 ± 0.1128 macro-F1). For AUC, MTGAF also showed the highest mean value (0.9297 ± 0.0437), followed by BrainGNN (0.9057 ± 0.0608) and BrainNetTF (0.8763 ± 0.0582). Compared with the traditional machine-learning classifiers, MTGAF showed numerically higher mean values across all reported metrics, including accuracy, precision, recall, macro-F1, and AUC.

Robustness analyses based on pooled cross-validation predictions yielded findings consistent with the primary performance estimates. For MTGAF, the pooled confusion matrix showed 42 true negatives and 46 true positives, with 6 false positives and 6 false negatives. Participant-level bootstrap analysis yielded 95% CIs of 0.8100–0.9400 for accuracy, 0.8095–0.9396 for macro-F1, and 0.8696–0.9770 for pooled AUC. Calibration measures included a Brier score of 0.1153 (95% CI, 0.0577–0.1802) and an expected calibration error of 0.1166 (95% CI, 0.0614–0.1870). Paired comparisons showed positive differences in accuracy and macro-F1 for MTGAF relative to BrainNetTF, BrainGNN, SVM, and Random Forest.

The single-modality ablation results indicated that the multiplex PC–SR–GCM representation provided the most stable overall classification performance. The full MTGAF model achieved higher test accuracy and macro-F1 than the PC-only, SR-only, and GCM-only variants (**Table 3**). The SR-only variant yielded the highest AUC among the single-modality models, but its accuracy and macro-F1 remained below those of the full model. Participant-level bootstrap 95% CIs for accuracy were 0.8100–0.9400 for the full MTGAF model, 0.7900–0.9300 for PC only, 0.7500–0.9000 for SR only, and 0.7600–0.9000 for GCM only. These intervals were consistent with the overall ablation pattern.

**Table 3:** Five-fold ablation results for the full MTGAF model and single-modality variants.

Variant	Accuracy	Precision	Recall	F1-score	AUC
**Full MTGAF**	**0.8800 ± 0.0678**	**0.8833 ± 0.0691**	**0.8814 ± 0.0678**	**0.8796 ± 0.0680**	0.9297 ± 0.0437
PC only	0.8600 ± 0.0490	0.8720 ± 0.0471	0.8563 ± 0.0524	0.8569 ± 0.0513	0.9176 ± 0.0267
SR only	0.8300 ± 0.0400	0.8417 ± 0.0420	0.8272 ± 0.0423	0.8268 ± 0.0416	**0.9458 ± 0.0350**
GCM only	0.8300 ± 0.0600	0.8357 ± 0.0624	0.8294 ± 0.0600	0.8287 ± 0.0601	0.9056 ± 0.0276

Bold formatting identifies the best-performing result for each metric.

Component ablation results are summarized in **Table 4**. Using the full MTGAF result as a reference (accuracy 0.8800 ± 0.0678, macro-F1 0.8796 ± 0.0680, and AUC 0.9297 ± 0.0437), the reduced-capacity MTGAF-small variant showed lower accuracy, macro-F1, and AUC, suggesting that the selected model capacity was beneficial for this task. Removing the graph-transformer block produced the lowest accuracy and macro-F1 among the component variants, supporting the role of long-range graph refinement. Removing adaptive fusion also reduced threshold-level classification performance, indicating that learned fusion weights were useful for combining modality-specific and interaction features. The variant without cross-modal attention retained relatively comparable accuracy but had a lower AUC than full MTGAF, suggesting that cross-modal attention contributed more to probability ranking and multimodal discrimination than to accuracy alone. Removing the disease-aware readout also reduced performance relative to the full model, supporting the use of disease-aware pooling.

**Table 4:** Exploratory component ablation results interpreted relative to the primary full MTGAF result.

Variant	Accuracy	Precision	Recall	F1-score	AUC
MTGAF-small	0.8100 ± 0.0583	0.8172 ± 0.0636	0.8083 ± 0.0596	0.8078 ± 0.0589	0.9078 ± 0.0703
Without graph transformer	0.8000 ± 0.0707	0.8024 ± 0.0714	0.8001 ± 0.0721	0.7988 ± 0.0716	0.9216 ± 0.0379
Without cross-modal attention	0.8600 ± 0.0374	0.8641 ± 0.0414	0.8594 ± 0.0379	0.8590 ± 0.0377	0.9237 ± 0.0276
Without adaptive fusion	0.8100 ± 0.0663	0.8172 ± 0.0639	0.8101 ± 0.0633	0.8085 ± 0.0661	0.9276 ± 0.0431
Without disease-aware readout	0.8500 ± 0.0447	0.8588 ± 0.0443	0.8494 ± 0.0450	0.8483 ± 0.0454	0.9216 ± 0.0410

### 3.4 Node importance analysis

**Figure 3:**
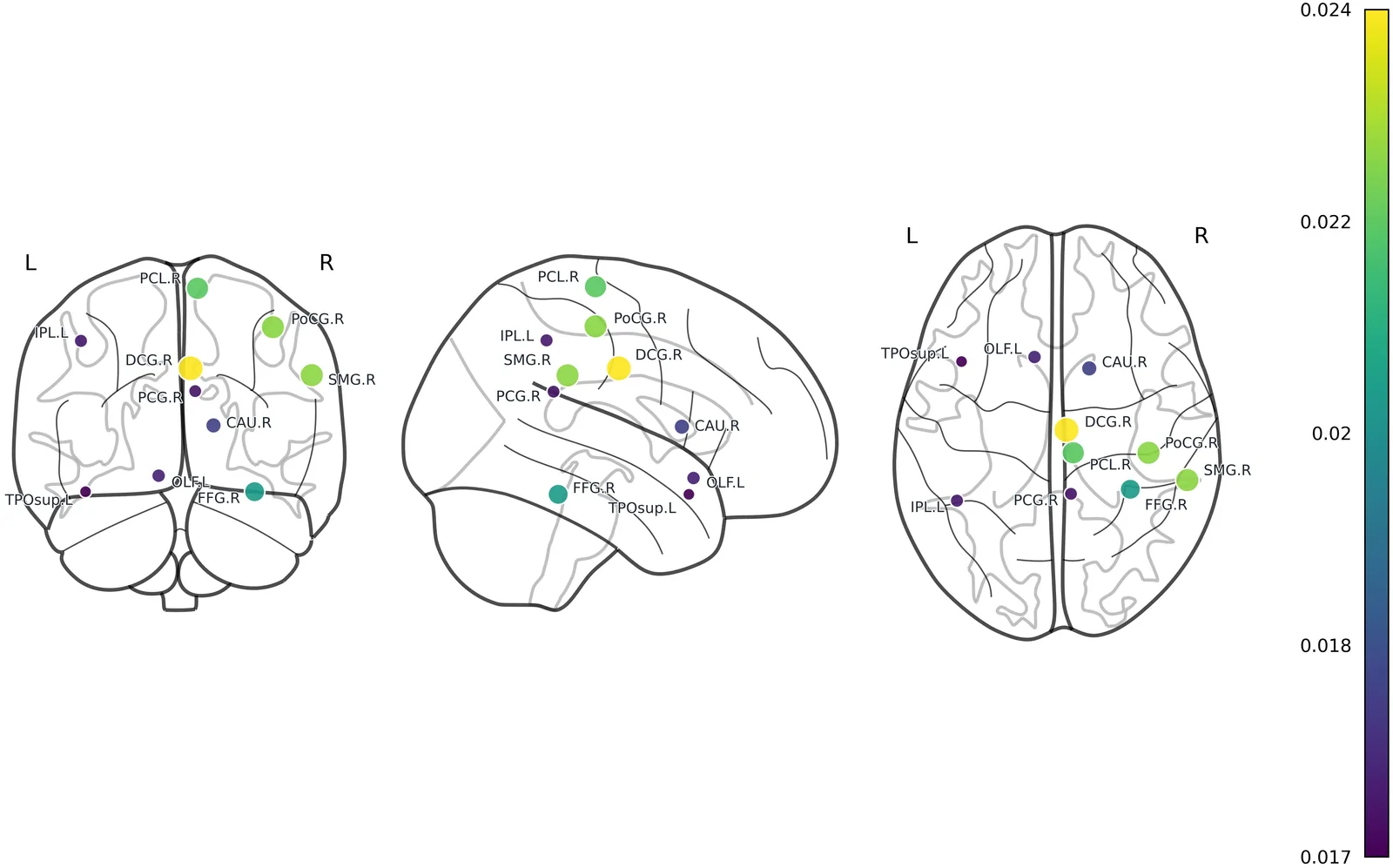
BrainNet visualization of ROI importance in the MTGAF model. Node color and size denote the relative importance of brain regions emphasized by the model. Region labels follow the AAL116 atlas.

To further characterize the regions emphasized by MTGAF, we performed an ROI importance analysis using the trained models. For each participant, the model explanation analysis estimated modality-specific ROI weights for the PC, SR, and GCM branches. We averaged these weights across CKD participants within their final test folds and then averaged the resulting ROI scores across the three connectivity views to obtain a single node-level importance score. Higher values therefore indicate model-assigned regional relevance in the disease-aware readout and fusion architecture, which is interpreted as a model-derived neuroimaging pattern. The top 10 nodes are summarized in **Table 5** and visualized in **Figure 3**.

**Table 5:** Top 10 brain nodes ranked by CKD ROI importance in the MTGAF model.

Rank	AAL116 label	Anatomical region	Putative network/system	Mean importance
1	DCG.R	Right median cingulate and paracingulate gyri	Cingulo-opercular/salience-related system	**0.023599**
2	SMG.R	Right supramarginal gyrus	Temporoparietal association system	0.022398
3	PoCG.R	Right postcentral gyrus	Primary somatosensory network	0.022388
4	PCL.R	Right paracentral lobule	Sensorimotor network	0.021824
5	FFG.R	Right fusiform gyrus	Ventral visual association system	0.020389
6	CAU.R	Right caudate nucleus	Frontostriatal executive circuit	0.017970
7	OLF.L	Left olfactory cortex	Orbitofrontal/olfactory association system	0.017244
8	IPL.L	Left inferior parietal lobule	Frontoparietal association system	0.017102
9	PCG.R	Right posterior cingulate gyrus	Default mode/posterior cingulate system	0.017010
10	TPOsup.L	Left superior temporal pole	Temporal association/limbic interface system	0.016571

Bold formatting identifies the highest mean ROI importance.

The highest mean node importance was observed in DCG.R, followed by SMG.R, PoCG.R, PCL.R, and FFG.R. Overall, 7 of the top 10 nodes were right-lateralized. At the systems level, the top nodes involved cingulate, temporoparietal, somatosensory, sensorimotor, ventral visual, frontostriatal, orbitofrontal/olfactory, frontoparietal, posterior cingulate, and temporal association regions.

## 4 Discussion

### 4.1 Principal findings

This study developed and evaluated MTGAF, a multiplex functional connectome graph transformer for individualized CKD-versus-control identification from rs-fMRI. The main contribution is that CKD-related brain-network information was modeled as a set of complementary connectome views rather than as a single functional connectivity matrix. In this cohort, MTGAF achieved the numerically highest test accuracy and macro-F1 among the evaluated classifiers and preserved balanced discrimination across sensitivity, specificity, macro-F1, and AUC. This performance pattern suggests good subject-level discrimination in the present cohort, and larger external studies can further assess transferability and relative advantages over comparator models. The full PC–SR–GCM model also achieved higher accuracy and macro-F1 that the PC-only, SR-only, and GCM-only variants, indicating that synchronous coupling, sparse dependency structure, and directed temporal association contributed nonidentical information to subject-level CKD identification.

The neuroanatomical findings further suggest that the classifier did not depend on a single regional abnormality. ROI importance involved cingulate, temporoparietal, sensorimotor, visual-association, frontostriatal, orbitofrontal/olfactory, frontoparietal, posterior cingulate, and temporal-association regions. This distributed pattern is consistent with the view that CKD-related brain dysfunction is expressed at the level of large-scale network organization. Bootstrap, calibration, confusion-matrix, and paired-comparison summaries provided additional support for the stability of this performance pattern.

### 4.2 CKD-related brain dysfunction as a network-level disorder

The present findings extend prior CKD neuroimaging work from group-level description toward individualized identification. Earlier rs-fMRI studies reported abnormal default mode network connectivity, altered structural-functional organization, and broader network disturbance in end-stage renal disease and dialysis cohorts (15–21, 24). More recent studies have emphasized dynamic functional connectivity abnormalities, interhemispheric disruption, neurovascular coupling changes, blood-oxygen-level-dependent cerebrospinal-fluid coupling, and imaging correlates of cognitive impairment (25, 26, 28–31, 58). Together, these studies indicate that CKD affects the coordination of distributed brain 387 systems rather than only one resting-state network.

This network-level interpretation is also biologically plausible. CKD is accompanied by vascular burden, metabolic and inflammatory stress, cerebrovascular dysregulation, and dialysis-related physiological instability, all of which may influence cerebral perfusion, neurovascular coupling, and long-range functional communication (9–11, 13, 14). Evidence from related systemic metabolic disorders, particularly type 2 diabetes with cognitive impairment, also highlights the value of rs-fMRI, machine learning, and multimodal imaging for studying disease-associated cognitive vulnerability (59, 60). The lower MMSE and MoCA scores observed in the CKD group are consistent with this systems-level burden and with reports that cognitive impairment is a common and clinically meaningful complication of CKD (2, 4, 5, 12). Thus, the classification task in this study is not merely a computational exercise; it is anchored in a recognized kidney-brain phenotype in which systemic renal disease is associated with altered brain-network organization and measurable group-level cognitive vulnerability. Accordingly, model interpretation is centered on CKD-versus-control identification rather than individual cognitive diagnosis.

### 4.3 Neuroscientific interpretation of ROI importance

The top-ranked ROIs provide a neurobiologically coherent, although model-derived, view of the discriminative pattern. The right median cingulate and paracingulate gyri were the most important regions. These areas lie within cingulate control and salience-related circuitry and are positioned to integrate internal state, cognitive control, and behavioral relevance. The right posterior cingulate gyrus adds a default-mode component, linking the present model to prior CKD studies that emphasized default mode network disruption (15, 16, 22). The simultaneous involvement of anterior/mid-cingulate and posterior cingulate territories suggests that CKD-related discrimination may reflect altered interaction between control, salience, and internally oriented networks rather than isolated default-mode impairment.

Temporoparietal and parietal association regions also contributed strongly. The right supramarginal gyrus and left inferior parietal lobule are implicated in attentional reorienting, multisensory integration, and higher-order association processing, functions that are relevant to the cognitive deficits observed in CKD. The right postcentral gyrus and right paracentral lobule indicate participation of somatosensory and sensorimotor systems. This finding is meaningful because CKD and dialysis impose persistent bodily, vascular, and metabolic stress; such systemic burden may be reflected not only in association networks but also in sensorimotor integration. The right caudate nucleus points to frontostriatal executive circuitry, a system important for cognitive flexibility, action selection, and goal-directed control. The fusiform gyrus, superior temporal pole, and olfactory/orbitofrontal region suggest that ventral visual, temporal-limbic, and orbitofrontal association pathways also contributed to the learned connectome signature.

Taken together, the ROI pattern supports a distributed-systems interpretation of CKD-related brain dysfunction. Rather than centering the discussion on a single abnormal node, the model highlighted regions that participate in cingulate-control, default-mode, frontostriatal, temporoparietal, sensorimotor, and temporal-limbic circuits. This interpretation is compatible with graph-theoretical and dynamic-connectivity studies showing that CKD can disturb both local and global functional organization (17, 19, 25, 26, 30). These ROI findings may be viewed as model-guided neurobiological patterns that merit further evaluation. Their clinical interpretation would be strengthened by independent replication, stability assessment, and direct association with cognitive, renal, vascular, and dialysis-related variables.

### 4.4 Methodological implications of multiplex connectome modeling

The performance profile supports the methodological rationale for multiplex graph fusion. PC, SR, and GCM represent different statistical views of the same rs-fMRI signals. PC captures undirected synchronous coupling, SR emphasizes sparse dependency relationships, and GCM represents directed temporal association. Because rs-fMRI Granger causality estimates are constrained by sampling rate, time-series length, preprocessing, and autoregressive-model assumptions, the GCM branch should be viewed as an additional directed temporal-association representation rather than as a causal mechanistic claim. For a systemic condition such as CKD, in which brain effects may arise from vascular, metabolic, inflammatory, and treatment-related mechanisms, it is unlikely that one connectivity estimator can fully characterize the disease-related network phenotype. The ablation results are consistent with this argument: each single view retained useful information, whereas the full multiplex model produced the strongest accuracy and macro-F1.

The higher AUC of the SR-only variant should be interpreted in this context. SR may provide a strong ranking signal by emphasizing sparse dependencies among brain regions, and this could explain why SR alone achieved high AUC. However, the full PC–SR–GCM model achieved higher accuracy and macro-F1, suggesting better threshold-level classification balance in this cohort. This metric-specific pattern suggests that sparse-dependency information was particularly discriminative for ranking, while adding PC and GCM information helped stabilize class assignment. Larger datasets can further clarify whether the AUC advantage of SR-only reflects sampling variability, modality-specific signal strength, or additional modality-specific variability.

This finding aligns with multimodal neuroimaging theory, which holds that disease information can be distributed across partially nonredundant representations (36–38). It also accords with network neuroscience accounts of brain disorders as alterations in both local segregation and long-range integration (61–63). Within MTGAF, graph convolution captured local neighborhood structure, graph-transformer refinement modeled longer-range inter-regional dependencies, disease-aware readout summarized regional evidence, and adaptive fusion combined modality-specific signals with pairwise cross-modal interactions. The component-ablation sensitivity analyses were directionally consistent with these design choices, particularly for graph-transformer refinement, adaptive fusion, disease-aware readout, and modality-specific encoding.

### 4.5 Clinical and translational relevance

The clinical relevance of this work lies in its connection between CKD, cognition, and individualized brain-network characterization. Cognitive impairment in CKD can affect medication adherence, dialysis self-management, shared decision-making, safety, and long-term outcomes (3, 5, 6, 8). A validated rs-fMRI-based model could eventually complement neuropsychological assessment by identifying patients who may warrant more detailed cognitive evaluation, neurological follow-up, or risk-focused care coordination. Such an approach would be particularly relevant in settings where cognitive changes are subtle, underrecognized, or difficult to separate from systemic illness burden.

The current model should therefore be viewed as a research-stage imaging-AI candidate for CKD-related brain-risk characterization. Its value lies in showing how multiplex connectome signatures can complement clinical assessment by translating rs-fMRI network information into subject-level CKD-versus-control characterization. Future studies can extend this work by assessing external validity, calibration, clinically meaningful decision thresholds, and incremental value beyond demographic, cognitive, renal, and vascular risk variables.

### 4.6 Limitations and future work

Several considerations frame the interpretation of these findings. First, the dataset was single-center, retrospective, modestly sized, and external validation was beyond the scope of the present analysis. These features make multicenter confirmation an important next step. Recent functional-connectome benchmarks also show the value of comparing graph deep learning architectures with simpler models under rigorous evaluation settings (64, 65). Second, the present analysis focused on binary CKD-versus-control classification rather than CKD stage, dialysis subtype, individual cognitive-impairment status, longitudinal cognitive decline, or treatment response. Third, ROI-importance weights are model-derived and intended to guide biological hypotheses; future stability testing, independent replication, and biological validation would further strengthen their interpretation. Fourth, the calibration estimates provide internal cross-validation summaries, and external cohorts can further refine probability calibration.

Future studies should validate MTGAF in larger multi-center cohorts and include richer renal, vascular, dialysis, and cognitive phenotyping. Future work should also test whether multiplex graph signatures can predict longitudinal cognitive outcomes or treatment-related changes in brain function. More broadly, recent biomedical and machine-learning studies highlight the value of combining heterogeneous clinical profiles for prognosis, adapting models across related biological domains, and improving recognition under limited target-domain examples (66–68). These directions are relevant to future CKD imaging-AI work because translation will benefit from models that generalize across scanners, sites, clinical phenotypes, and external cohorts. Future external studies can also incorporate explicit uncertainty estimation, which is increasingly emphasized in medical-imaging AI when translating model outputs into probabilistic interpretation (69). Prospective external validation would be an important step toward clinical decision-support research.

### 4.7 Conclusion

MTGAF demonstrated strong single-center performance for subject-level CKD-versus-control identification from rs-fMRI and improved accuracy and macro-F1 over single-connectivity variants. The findings support the view that CKD-related cerebral involvement may be expressed as a distributed multiplex connectome signature involving cingulate-control, default-mode, temporoparietal, sensorimotor, frontostriatal, and temporal-limbic systems. By jointly modeling synchronous coupling, sparse dependency structure, and directed temporal association, MTGAF provides a research-stage network-neuroscience framework for individualized brain-risk characterization in dialysis-treated CKD. Larger external validation, richer clinical phenotyping, calibration assessment, and pre-specified model-comparison testing will be important for advancing this framework toward clinical decision-support research.

## Data Availability

The data analyzed in this study consist of de-identified secondary rs-fMRI time-series and connectivity matrices derived from the source cohort. Access to the underlying imaging data is subject to source-cohort governance and institutional permission. Where permitted by applicable data-use restrictions, de-identified derived analysis materials may be made available by the corresponding author upon reasonable request.
